# Gut microbiota as a therapeutic target in sepsis-induced multi-organ dysfunction: a review

**DOI:** 10.3389/fcimb.2026.1823125

**Published:** 2026-06-19

**Authors:** Xinlan Zhao, Tiangang Ma, Jinyan Yu, Yanbing Hu

**Affiliations:** 1 Department of Respiratory and Critical Care Medicine, The Second Hospital of Jilin University, Changchun, Jilin, China; 2 Department of Ultrasound Medicine Department, The Second Hospital of Jilin University, Changchun, Jilin, China

**Keywords:** gut microbiota, inflammatory responses, intestinal barrier disruption, multi-organ dysfunction, organ damage

## Abstract

Sepsis and the resulting multiple organ dysfunction carry a high mortality rate, and effective treatment remains lacking owing to the condition’s heterogeneity. The translocation of gut microbiota plays an essential role in the occurrence and development of sepsis and organ dysfunction. Uncontrolled inflammatory responses in sepsis, together with antibiotic use during treatment, lead to gut microbiota translocation. This in turn promotes sepsis progression and organ dysfunction by altering microbial metabolite production and causing immune dysregulation. Drawing on literature from databases such as PubMed and Web of Science, this review summarizes the interactions between intestinal microbiota translocation, sepsis, and organ dysfunction, aiming to identify new targets for the prevention and treatment of sepsis.

## 1 Introduction

Sepsis, a life-threatening organ dysfunction resulting from a dysregulated host response to infection, remains a major challenge in global public health. The body faces a dual threat when the host’s local immune regulation against infection fails. Pathogens and their structural components—such as lipopolysaccharide (LPS), peptidoglycan, and other pathogen-associated molecular patterns (PAMPs)—as well as endogenous danger signals released by damaged tissues, including high-mobility group box 1 and other damage-associated molecular patterns (DAMPs), ignite a systemic inflammatory response that leads to the development and progression of multiple organ dysfunction.

The gut ecosystem is closely associated with the immune system. Sepsis can disrupt gut microbiota homeostasis and compromise the integrity of the intestinal mucosal barrier, facilitating the translocation of bacteria to extraintestinal tissues. This translocation consequently triggers systemic infections and exacerbates sepsis progression (Mittal and Coopersmith, 2014). Furthermore, the gut microbiome contributes to the pathophysiology of sepsis and organ damage through complex immune regulation and metabolic pathways, and dysbiosis negatively affects both the progression and prognosis of the condition (Haak and Wiersinga, 2017). Although the interplay between the gut microbiota and sepsis has been discussed in several reviews, a systematic synthesis that explicitly adopts the “gut–organ axis”framework to delineate how microbiota-driven signals concurrently affect multiple distant organs is still lacking. The present review fills this gap by comprehensively examining the interactions among gut microbiota translocation, sepsis, and organ dysfunction through the lens of this axis. Therefore, maintaining gut microbiota homeostasis could be an important target for the treatment of sepsis and organ damage. This review systematically summarizes the latest research progress in this field to provide a novel theoretical basis and intervention strategies for the precise diagnosis and treatment of sepsis and for the management of multi-organ damage.

## 2 Mechanism of sepsis-induced intestinal barrier disruption

The intestinal barrier maintains intestinal microbiota homeostasis and indirectly regulates systemic stability. This barrier mainly comprises the physical epithelial layer, the mucus layer, and the biological barrier. The intestinal epithelial cells form a single layer with a large surface area of approximately 32 square meters. Adjacent epithelial cells are closely connected via tight junctions (TJs), desmosomes, and adherens junctions, forming a compact and functionally complete barrier. The small intestinal endothelium is primarily distributed within capillaries and lymphatic vessels in the lamina propria and is responsible for substance exchange. These cell barriers block harmful substances while selectively regulating the absorption of essential nutrients (Horowitz et al., 2023; Oami et al., 2025).

The intestinal biological barrier consists of bacteria, viruses, and fungi, totaling approximately 40 trillion cells. This microbiota actively regulates the host’s physiological functions through direct and indirect pathways, playing a crucial role in maintaining the health of almost all organ systems (Sender et al., 2016). Specifically, it protects the intestinal epithelial barrier, resists pathogens, promotes nutrient absorption, and regulates digestion, metabolism, and neuro-immune processes (Li et al., 2021). When severe diseases damage intestinal barrier function, microorganisms and their products can enter the systemic circulation via TJ-dependent and TJ-independent pathways, thereby further disrupting an already imbalanced immune system.

### 2.1 Effect of sepsis on the cell barrier

The intestine is the largest immune organ and bacterial reservoir in the body, and the integrity of its barrier structure serves as a crucial line of defense in maintaining homeostasis. Sepsis induces endothelial leakage, microthrombi formation, epithelial cell death, and the disintegration of intercellular connections, thereby destroying the intestinal barrier. The intestinal microbiota and endotoxins enter the portal circulation and lymphatic system through the damaged intestinal barrier, and subsequently translocate throughout the body, exacerbating systemic endotoxemia, immune imbalance, coagulation disorders, inflammation, and damage in distant organs, such as the lungs and liver.

The microvascular endothelial cells of the intestine represent an initial physical line of defense against bacterial and toxin translocation. During sepsis, high levels of inflammatory factors, including tumor necrosis factor-alpha (TNF-α) and interleukin (IL)-1β, are released, activating signaling pathways such as NF-κB. The activation of these pathways leads to cytoskeletal remodeling, endothelial cell contraction, and the disintegration of the adherens junctions formed by vascular endothelial cadherin, resulting in gaps between cells and a 35% increase in vascular permeability. In septic patients, increased levels of soluble Tie2 have been associated with organ failure and mortality (Wegmann et al., 2025), and animal studies have shown that Tie2 downregulation leads to capillary leakage. Combined, these findings suggest a critical role for Tie2 in sepsis-induced endothelial barrier disruption.

Endothelial cell activation and damage disrupt the local balance that promotes anticoagulation. Overactivation of the Toll-like receptor 4 (TLR4) and NLRP3 inflammatory pathways amplifies inflammation and impairs endothelial anticoagulant function. This overactivation promotes platelet adhesion, aggregation, and the formation of neutrophil extracellular traps (NETs), triggering microvascular immunothrombosis. This results in insufficient intestinal tissue perfusion, ischemia, and hypoxia, thereby further aggravating intestinal barrier damage and forming a vicious cycle of “leakage–ischemia–more damage” (Soriano et al., 2005; Swanson et al., 2019; Joffre et al., 2020; Shi et al., 2021; Aklilu et al., 2025) These findings are based primarily on murine sepsis models and *in vitro* studies (Swanson et al., 2019; Joffre et al., 2020; Shi et al., 2021; Aklilu et al., 2025). Clinical evidence for NETs and microvascular immunothrombosis in human sepsis is accumulating but remains limited to observational studies. For instance, recent clinical studies suggest that TLR4 antagonism has failed to improve outcomes in large trials, indicating that redundancy or compensatory mechanisms may limit the translational relevance of single-target strategies (Zhu et al., 2026).

The epithelial layer is the solid core of the intestinal barrier. In sepsis, endotoxins, bacterial metabolites—such as acrolein—and the inflammatory environment induce excessive apoptosis of intestinal epithelial cells, resulting in the loss of functional barrier integrity (Chen et al., 2017; Park et al., 2018; Charitos et al., 2025). Necroptosis mediated by the RIPK1/RIPK3/MLKL pathway is a key mechanism driving rapid barrier rupture (Laurien et al., 2020). Additionally, pyroptosis plays an important role in damaging epithelial cell barrier function. PAMPs and DAMPs activate NLRP3 and other inflammasomes, cleaving Gasdermin D and forming pores on the cell membrane, which leads to osmotic cell lysis. The release of high levels of IL-1β and IL-18 dramatically amplifies local inflammation (Downs et al., 2020; Zhang et al., 2024). Damaged epithelial cells actively release cytokines such as IL-33, thereby activating type 2 immune responses and shaping a pro-inflammatory local microenvironment (Akama et al., 2024). These pathways are interconnected, exacerbating epithelial cell damage and driving increased cytokine and mediator release, thereby forming a vicious cycle.

The dissociation of intercellular connection complexes is the direct molecular basis for barrier dysfunction. TJs and adherens junctions form the basis of the cell barrier, and their integrity is maintained by key proteins, such as zonula occludens-1 (ZO-1), occludin, and cadherins. Inflammatory factors released during sepsis target these proteins. TNF-α downregulates the expression of the *TPJ1* gene encoding the TJ protein ZO-1 and induces its internalization from the plasma membrane to the cytoplasm, thereby disrupting the integrity of the TJ (Barros Ferreira et al., 2024). Simultaneously, TNF-α also induces cell elongation, cytoskeletal remodeling, stress fiber formation, and phosphorylation of the myosin light chain, triggering myosin-actin contraction and opening TJs (Jin and Blikslager, 2020). TNF-α and interferon-gamma (IFN-γ) have similar biological activities; however, IFN-γ does not target TJ proteins but specifically acts on adherens junctions. In particular, IFN-γ causes the disordered distribution of vascular endothelial (VE) cadherin; however, it does not affect the distribution of ZO-1 (Colás-Algora et al., 2020). Short-term (4-hour) induction of IFN-γ leads to a discontinuous distribution of VE cadherin and β-catenin, resulting in significant intercellular gaps and increased barrier permeability (Ng et al., 2018); long-term IFN treatment causes a linear distribution of these two proteins and significantly downregulates the expression of adhesion proteins, including membrane and cytoskeleton-associated proteins (Ng et al., 2023; Ng et al., 2025).

### 2.2 Effect of sepsis on biological barriers

The microbiota can undergo homeostatic disruption owing to inflammatory immune factors, specific metabolites, antibiotic use, and bacterial translocation following barrier damage. The host immune system responds to infection or injury by releasing inflammatory factors that alter the intestinal environment—through increased permeability, altered pH, and oxidative stress—thereby inhibiting the growth of beneficial bacteria, including *Lactobacillus* and *Bifidobacterium*, and promoting the proliferation of potential pathogens like *Escherichia coli* and *Salmonella* (Bao et al., 2022). Conversely, short-chain fatty acids (SCFAs), such as butyrate and propionate, as well as bile acids and polyamines produced by microbial metabolism, play a crucial role in maintaining homeostasis (Wang et al., 2024). For example, SCFAs inhibit inflammation by regulating the differentiation of T regulatory (Treg) cells (Smith et al., 2013). However, this metabolic profile often shifts when the microbiota is disrupted; specifically, the depletion of these beneficial metabolites can act directly as signaling molecules, affecting host gene expression and the immune response.

**Figure 1:**
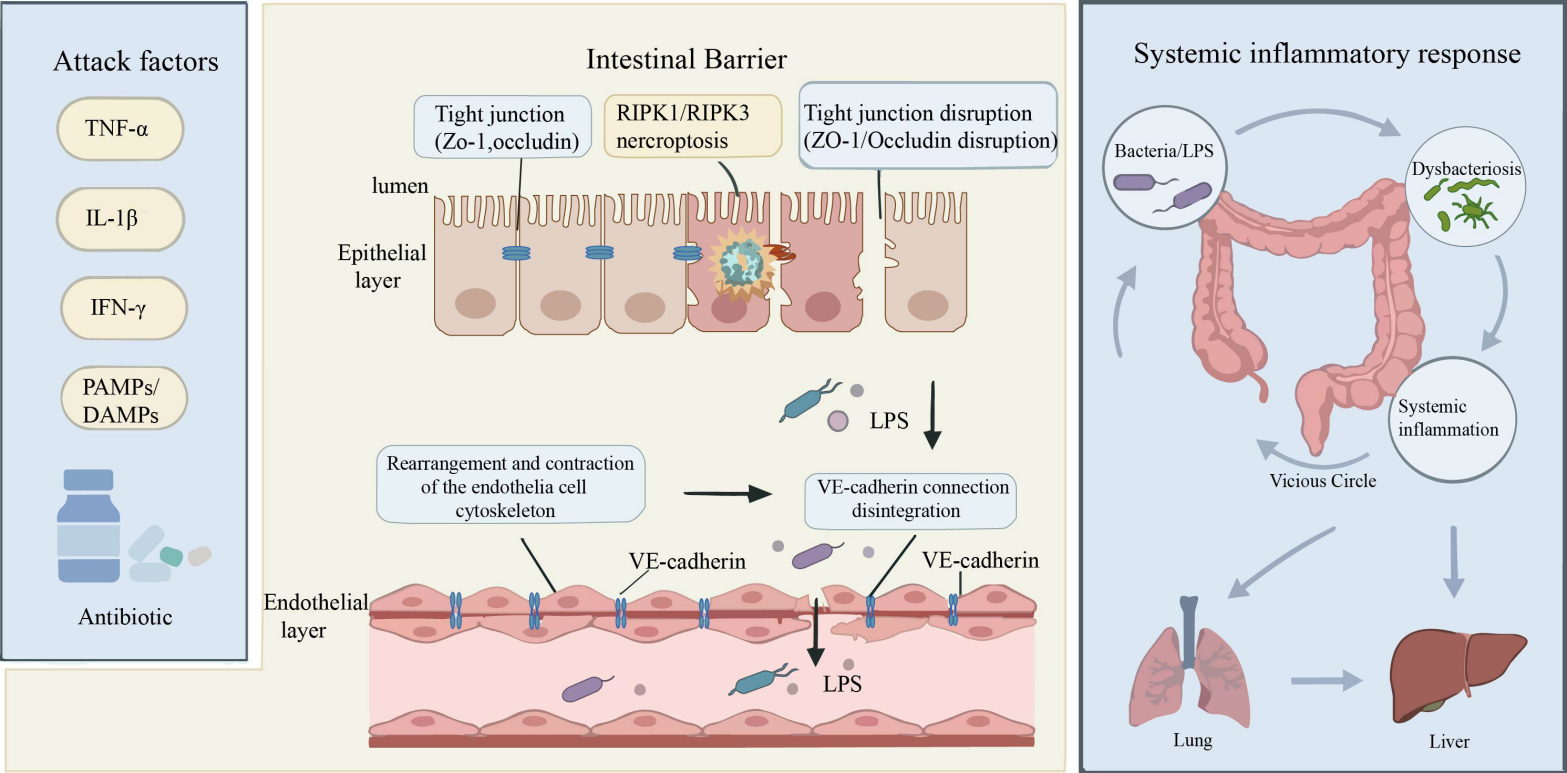
The disruption of intestinal barrier in sepsis and its impact on multiple systems. When sepsis manifests, inflammatory factors such as TNF-α, IFN-γ, IL-1β, and PAMPs and DAMPs are the key factors responsible for damaging intestinal barrier. TNF-α downregulates tight junction proteins at the genetic level; IL-1β activates the NF-KB immune pathway, triggering the contraction and reorganization of the endothelial cell cytoskeleton; IFN-γ causes disintegration of VE-cadherin, and leads to damage of adherens junctions. RIPK1/RIPK3/MLKL pathway regulates necroptosis-driven barrier disruption. Based on the nature of destruction of the intestinal barrier, gut microbiota translocation or disorder occurs, leading to local or multi-system inflammatory responses. These responses trigger an inflammatory storm, further disrupting the balance of the microbial system. Eventually, this may lead to damage of distant organs such as lungs and liver. Besides, the abuse of antibiotics further destroys the gut microbiota. Created with Adobe illustrator.

Multiple antibiotics are often used in combination to treat sepsis; however, the risk of drug-induced dysbiosis in the gut microbiota increases markedly owing to the widespread use of these high-dose regimens. Some clinical trials have reported a significant correlation between gut microbiota dysbiosis and multi-drug combination therapy. Observations recorded 48 hours after treatment have revealed a structural shift in the microbiota, and these alterations in the abundance of antibiotic resistance genes can persist for up to 12 months (Ticinesi et al., 2017; Reyman et al., 2022). Furthermore, intestinal barrier damage and loss of integrity facilitate bacterial translocation. Once the barrier is compromised, bacteria and their products, such as LPS, can penetrate the intestinal wall, activating local and systemic immune responses. This triggers innate immunity through the TLR signaling pathway, leading to a cytokine storm and further disrupting microbial homeostasis. A vicious cycle forms between barrier damage, bacterial translocation, and the inflammatory response: microbiota dysbiosis increases barrier permeability, while displaced bacteria exacerbate inflammation, further damaging the microbiota (**Figure 1**).

## 3 Effect of gut microbiota dysbiosis on various organs and systems in sepsis

### 3.1 Effect of gut microbiota dysbiosis on the central nervous system

A complex bidirectional communication network exists between the gut microbiota and the central nervous system. In 2023, the vagus nerve-mediated neural pathway of the gut–brain axis was confirmed *in vivo* for the first time using high-resolution microscopy imaging (Wang et al., 2023; Wang et al., 2024). Consequently, any disruption to this intestinal microbiota homeostasis can contribute to central nervous system disorders and demyelinating lesions, such as multiple sclerosis, anxiety, depression, and Alzheimer’s disease (Wang et al., 2024; Ivanova et al., 2025; Zhang et al., 2025). During sepsis, this dysregulation of the intestinal microbiota ultimately affects the central nervous system by modulating neurotransmitter signaling, metabolic pathways, and immune function.

#### 3.1.1 Gut microbiota affects central nervous system function through neurotransmitter synthesis and metabolism

The gut microbiota influences the synthesis and metabolism of neurotransmitters, including dopamine, serotonin, and gamma-aminobutyric acid (GABA). These processes become disordered during sepsis-induced gut dysbiosis. Although the link between the gut microbiota and catecholamine metabolism has been reported in Parkinson’s disease (Asano et al., 2012), it is not yet confirmed whether the same mechanism operates in sepsis. In sepsis, the rapid onset of systemic inflammation and antibiotic-induced dysbiosis may lead to distinct metabolic changes that require direct investigation. Simultaneously, microbiota dysbiosis-induced dysfunction of the 5-hydroxytryptamine (serotonin) system can transmit signals via the vagus nerve to the brain stria terminalis, thereby affecting emotions, cognition, and stress behaviors.

#### 3.1.2 Effect of intestinal metabolites on the central nervous system

Beneficial gut bacteria produce SCFAs, such as butyrate, propionate, and acetate, through the fermentation of dietary fiber. Under physiological conditions, SCFAs exert multi-level protective effects. In the intestinal environment, butyrate stimulates intestinal epithelial cells to secrete mucin and upregulates the expression of TJ proteins, such as occludin and ZO-1, thereby enhancing the physical barrier function of the intestinal epithelium and limiting the translocation of bacteria and endotoxins like LPS (Barcelo et al., 2000; Wang et al., 2024). Furthermore, in a mouse model of sepsis-associated encephalopathy, butyrate supplementation was shown to upregulate tight junction proteins in the blood-brain barrier and improve neurological outcomes (Liu et al., 2021); however, clinical validation in septic patients is lacking.

SCFAs also possess strong immunomodulatory capabilities. For example, they activate receptors on epithelial cells—such as GPR43—and induce the production of antimicrobial peptides, including RegIIIγ, further consolidating local defenses (Zhao et al., 2018; Welathanthree et al., 2025). More importantly, butyrate promotes the differentiation and function of Tregs in the colon, thereby directly inhibiting excessive inflammatory responses, reducing IL-6 and TNF-α production, and mitigating their effects on the central nervous system (Dalile et al., 2019).In addition to these direct effects, SCFAs influence neuroinflammation via the aryl hydrocarbon receptor pathway and modulate the HPA axis, as discussed below.

**Figure 2:**
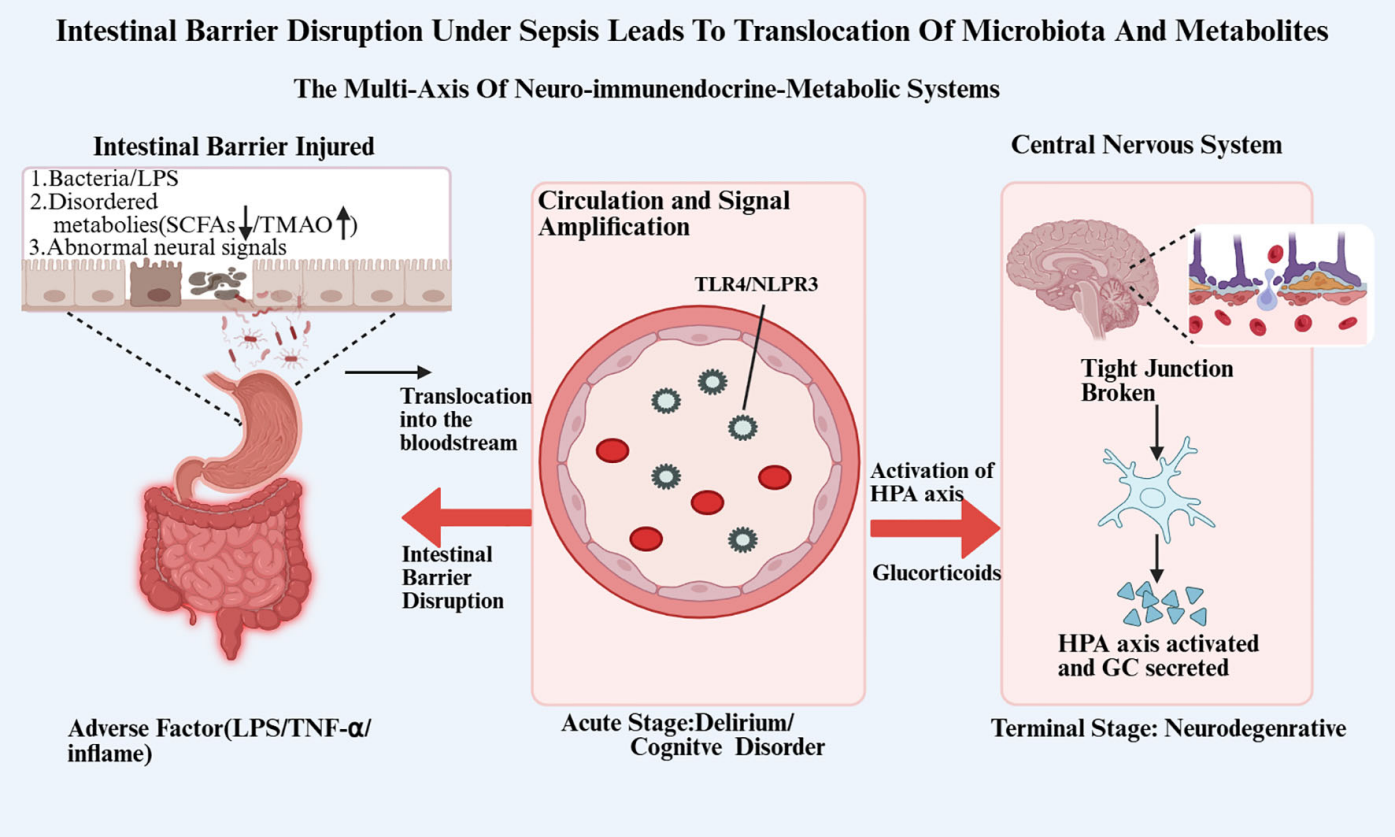
Intestinal barrier disruption under sepsis leads to translocation of microbiota and metabolities. After the intestinal barrier is impaired, it impacts the nervous system, causing disorders of bacteria and LPS, bacterial metabolites, and abnormal neural signals entering the blood. It activates the immune pathway to amplify the inflammatory response, further aggravating intestinal damage; and abnormal neural signals entering the blood can lead to cognitive impairment. Inflammatory factors destroy the blood-brain barrier, activate microglia, and activate the hypothalamic-pituitary-adrenal axis, leading to secretion of glucocorticoids and ultimately resulting in neurodegeneration. Created with BioRender.com.

The production of SCFAs decreases or disappears rapidly during sepsis due to severe microbiota dysbiosis, thereby weakening the protective mechanisms described above. Elevated levels of pro-inflammatory cytokines, such as IL-1β and TNF-α, in the circulation can activate the hypothalamic–pituitary–adrenal axis, leading to the continuous secretion of glucocorticoids. These glucocorticoids inhibit immunity and exacerbate intestinal permeability, forming a vicious cycle of “stress–intestinal leak–immune activation–stronger stress,” which exposes the nervous system to a persistently inflammatory environment (Van Wyngene et al., 2021; Pan et al., 2025) (**Figure 2**).

### 3.2 Effect of gut microbiota dysbiosis on the circulatory system during sepsis

The involvement of the cardiovascular system is a key factor determining the prognosis of sepsis-associated multi-organ dysfunction. Severe intestinal microbiota dysregulation and altered metabolites are not only a consequence of sepsis-induced damage but also contribute to cardiomyocyte injury, disrupt cardiac electromechanical function, and induce thrombosis, leading to myocardial injury and microcirculatory disorders. First, the entry of endotoxins, such as LPS, into the bloodstream resulting from intestinal barrier disruption causes direct and indirect damage to the myocardium. During sepsis, circulating LPS activates Toll-like receptors on the surface of immune cells—such as macrophages—triggering a cascade of pathways, including the NLRP3 inflammasome, which leads to a cytokine storm characterized by elevated TNF-α and IL-1β. These inflammatory factors inhibit myocardial cell contraction, disrupt intracellular calcium homeostasis, and reduce myocardial contractility (Zhuang et al., 2022; Yao et al., 2024). LPS can also induce cardiomyocyte apoptosis, increase mitochondrial reactive oxygen species production, and promote myocardial fibrosis and pathological hypertrophy, thereby exacerbating cardiac structural and functional damage through multiple mechanisms.

Second, SCFA dysregulation directly impacts cardiac function. During sepsis-associated microbiota dysbiosis, SCFA production decreases, thereby weakening the heart’s protective mechanisms. For instance, acetic acid supplementation improves cardiac contractility and inhibits myocardial hypertrophy (Marques et al., 2017). A 2025 study found that a group treated with SCFAs—including acetate, propionate, and butyrate—had a significantly attenuated increase in blood pressure compared to the control group (R et al., 2025). Because these findings were derived from a hypertensive animal model rather than a sepsis model, their extrapolation to sepsis should be cautious; the cardiovascular pathophysiology in sepsis (e.g., distributive shock, myocardial depression) differs fundamentally from chronic hypertension. Direct evidence for SCFAs in septic cardiomyopathy is still limited. Conversely, butyrate inhibits the contraction ability of cardiomyocytes by activating the GPR41 receptor on these cells and inhibiting the activity of the sarcoplasmic reticulum calcium pump (Yao et al., 2024). This finding suggests that different metabolites have a nuanced, “double-edged sword” effect on cardiac regulation.

Finally, intestinal microbiota dysregulation exacerbates microcirculatory disorders in sepsis by promoting immunothrombosis and affecting vascular integrity. LPS and microbiota-derived metabolites, such as trimethylamine N-oxide (TMAO), are pivotal drivers of these microcirculatory disorders. LPS can activate neutrophils and induce NET release. This structure provides a scaffold for platelet adhesion and aggregation and activates the coagulation pathway, forming immune thrombi in microvessels. TMAO also activates vascular endothelial cells, increasing the expression of E-selectin and intercellular adhesion molecule-1, promoting platelet hyperreactivity and white blood cell adhesion, and initiating procoagulant and atherosclerotic-like signaling pathways, which further exacerbate microcirculation failure (Seldin et al., 2016; Papa et al., 2023). Furthermore, TNF-α-induced endothelial cell apoptosis damages the integrity of the microvascular structure, whereas certain protective signals, such as the activation of transforming growth factor-beta-activated kinase 1 (TAK1), may play a compensatory role in this process (Naito et al., 2019; Pontarollo et al., 2023).

### 3.3 Role of gut microbiota in sepsis-induced acute lung injury

Recently, the gut–lung axis has received increasing attention, focusing primarily on the gut microbiota, the immune system, and the bidirectional interactions between the gut and the lungs. During sepsis, uncontrolled inflammatory responses, oxidative stress, and other factors can lead to pulmonary inflammation and barrier dysfunction, resulting in acute lung injury and potentially acute respiratory distress syndrome.

#### 3.3.1 Effect of gut microbiota translocation on the lungs

A study of acute lung injury caused by sepsis reported the presence of *Bacteroides* spp. and a disrupted lung microbiota in the bronchoalveolar lavage fluid of critically ill patients, along with elevated TNF-α levels, providing evidence for the translocation of intestinal microbiota to the lungs (Dickson et al., 2016). For patients with underlying conditions, such as chronic obstructive pulmonary disease (COPD), the translocation of intestinal bacteria and their metabolites is highly likely to occur, which can promote COPD progression by spreading bacterial toxins through systemic inflammatory responses (Zhong et al., 2015; Cai et al., 2020). Conversely, restoring the homeostasis of the intestinal microbiota can alleviate inflammatory lung injury. Yao et al. discovered a bacterial strain, named Δ PESI, that can produce IL-1Ra via outer membrane vesicles. Following treatment, this strain can colonize the intestinal mucosa, enhance barrier function, inhibit the inflammatory cascade, regulate lung macrophage pyroptosis, and maintain microbiota homeostasis (Yao et al., 2025).

#### 3.3.2 Effects of intestinal microbial metabolites on the lungs

**Figure 3:**
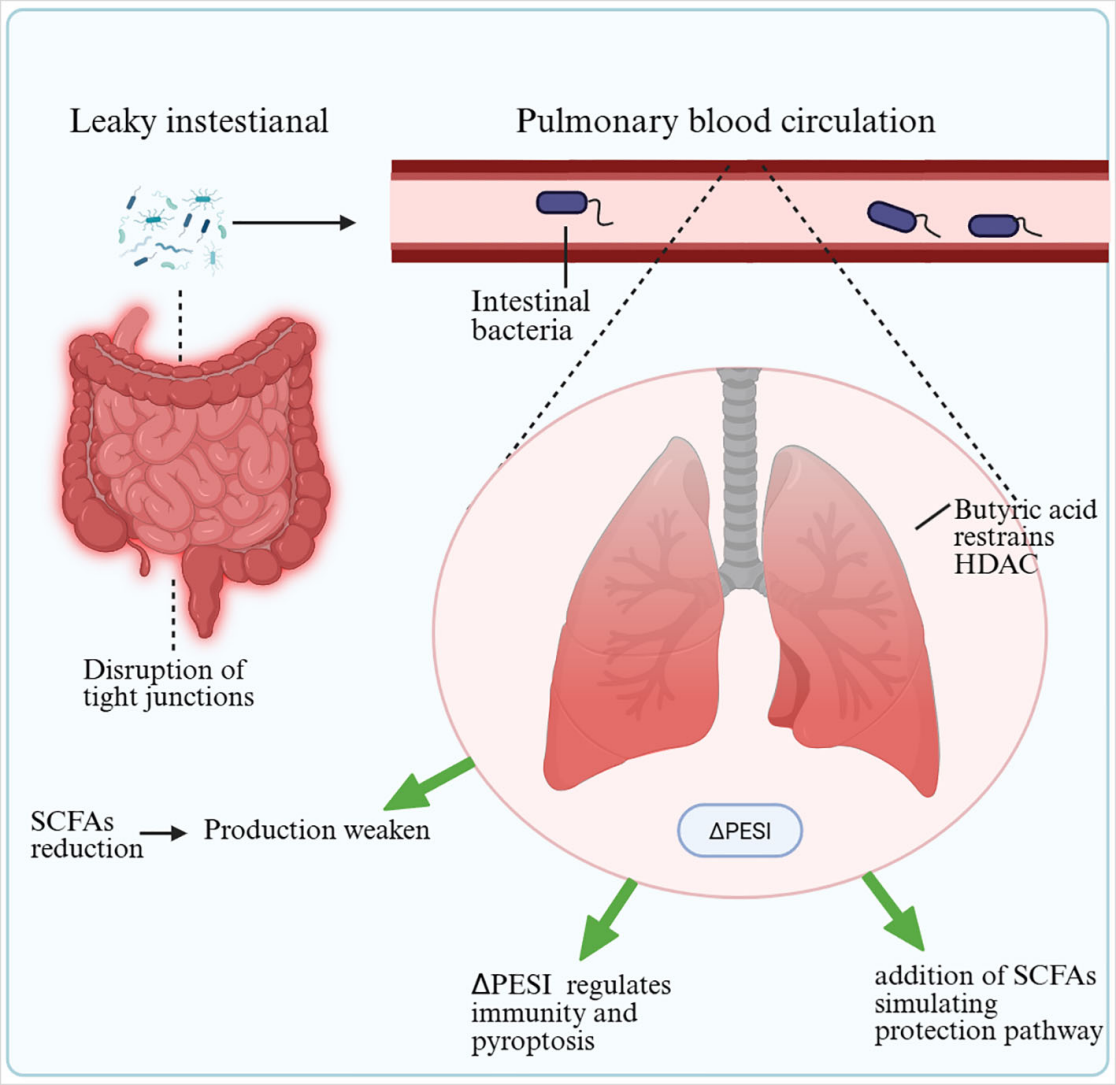
The impact of gut microbiota imbalance on the lungs. Translocated intestinal bacteria can be transplanted to the lungs through the bloodstream, which increases the risk of COPD progression in patients. SCFAs can have a positive effect on the lungs. Butyric acid enhances the epithelial barrier by inhibiting HDAC and regulates anti-inflammatory genes. SCFAs inhibit inflammatory responses by activating GPR41; exogenous addition of engineered bacteria Δ PESI and supplementation of SCFAs can simulate protective pathways. Created with BioRender.com.

Gut microbial metabolites regulate lung inflammation through epigenetic mechanisms: they provide acetyl-CoA for histone acetylation and act as HDAC inhibitors (Ney et al., 2023). For example, butyrate has been shown to inhibit NF-κB activation in lung epithelial cells and reduce airway inflammation in COPD models (Zhao et al., 2023); however, whether these epigenetic effects are directly operative in sepsis-induced acute lung injury requires further investigation (**Figure 3**).

### 3.4 Role of gut microbiota in sepsis-induced damage to the digestive system

Uncontrolled inflammatory responses and microcirculation disorders typically trigger sepsis-induced intestinal damage. Gut-associated lymphoid tissue (GALT) is hyperactive, producing large amounts of inflammatory mediators and exacerbating local and systemic inflammatory storms. Simultaneously, pathogenic bacteria (such as certain anaerobes) grow rapidly, significantly eliminating beneficial bacteria, including obligate anaerobes, and altering the metabolic capacity of the microbial community. During sepsis, intestinal inflammatory mediators, such as TNF-α and IL-6, inhibit smooth muscle contraction and nerve function (Castro et al., 2023). These intestinal motility disorders result from this surge of inflammatory factors when intestinal microbiota homeostasis is disrupted. Furthermore, TNF-α and IL-6 levels increase significantly following endotoxin injection during sepsis simulations, neutralizing either cytokine can partially restore gastrointestinal motility. Consequently, severe intestinal diseases allow bacteria and toxins to migrate into the mesenteric lymph nodes and portal venous circulation from the intestinal cavity, triggering liver dysfunction. Disruption of the intestinal microbiota thus exacerbates sepsis-induced damage to the digestive system.

#### 3.4.1 Role of metabolites in the gut during sepsis

The gut microbiota is crucial to maintaining epithelial barrier integrity by competing for ecological niches with pathogens *in situ* and producing metabolites with host regulatory activity. For example, butyrate and propionate can stimulate the expression of TJ proteins such as ZO-1 and occludin, thereby enhancing intercellular connections. Changes in the structure of the microbiota alter its antigen composition, leading to the activation of abnormal immune pathways and GALT, which causes excessive local inflammatory responses such as the Th17 response (Mörbe et al., 2021). Notably, the dual effects of butyrate—protective at low concentrations but detrimental at high doses—highlight the challenge of translating these findings to sepsis, where luminal concentrations of SCFAs are highly variable (Piccioni et al., 2024). Moreover, most studies cited (e.g., in elderly mice) were not performed directly in sepsis models; whether these effects can be replicated in septic patients with a dysbiotic microbiome remains to be tested. Future studies should therefore examine dose-response relationships and the impact of co-existing inflammatory conditions on SCFA efficacy.

#### 3.4.2 Role of gut microbiota in the liver during sepsis

Microbial dysbiosis and intestinal barrier damage, in conjunction, promote bacterial translocation and systemic endotoxin release-induced liver injury (SLI). The TLR4/NF-κB axis is the core immune pathway of SLI, and RPPS exerts anti-inflammatory effects by targeting this pathway to improve SLI. The glycosaminoglycan RPPS restores intestinal integrity primarily by upregulating occludin expression, thereby reducing bacterial translocation and the endotoxin load to the liver. After intestinal homeostasis is restored, beneficial metabolites such as butyrate increase, enhancing intestinal barrier function through G protein-coupled receptor signaling and inhibiting NF-κB activation, which subsequently reduces systemic inflammation (Zamyatina and Heine, 2020; Wang et al., 2025). In addition to mitigating intestinal inflammation and balancing Th17/Treg immune function, SCFAs can circulate in the portal vein via transmembrane transport mediated by monocarboxylate transporter 1, thereby regulating liver lipid metabolism pathways.

### 3.5 Effect of sepsis on the circulatory system

During sepsis, intestinal mucosal barrier function is severely impaired, leading to the translocation of intestinal microorganisms and their products into the systemic circulation. The components of the translocated microbiota and their metabolites impact the cellular components and coagulation function of the circulatory system through direct action and indirect immune activation.

#### 3.5.1 Effects of microbiota translocation on circulatory system components

Translocated intestinal bacteria (such as gram-negative bacteria) and their components (such as LPS) induce mononuclear and macrophage cells to release TNF-α and IL-1β, which directly activates platelets and facilitates their aggregation and adhesion (Aklilu et al., 2025). This activated state consequently enables them to actively participate in the subsequent pathological thrombosis process.

LPS in the circulation can strongly induce IL-6 production, which upregulates hepcidin expression by activating the JAK-STAT and other signaling pathways. High hepcidin levels inhibit intestinal iron release from macrophages, leading to serum iron deficiency. This “functional iron deficiency” severely hinders hemoglobin synthesis in erythroid precursor cells, thereby causing or exacerbating anemia (Nemeth et al., 2004; Marques et al., 2022).

Long-term severe inflammation and metabolic disorders can jeopardize hematopoietic function in the bone marrow. On the one hand, excessive inflammatory signals inhibit the proliferation of hematopoietic stem and progenitor cells and induce their apoptosis. On the other hand, the depletion of SCFAs like butyrate during sepsis weakens their anti-inflammatory and epigenetic support of the bone marrow microenvironment. This disruption can lead to bone marrow compensatory failure and exacerbate pancytopenia, characterized by a profound reduction in circulating immune cells, particularly lymphocytes, such as B and CD4+ T cells.

#### 3.5.2 Effects of microbiota translocation on the coagulation system

Microbiota translocation primarily triggers the hypercoagulable state and disseminated intravascular coagulation in sepsis. LPS from the intestine entering the blood activates TLR4 on the surface of immune cells, such as monocytes and macrophages, thereby inducing the upregulation of tissue factor (TF). TF is a key initiating factor in the extrinsic coagulation pathway, and its high expression directly leads to enhanced thrombin generation, activating the coagulation system (He et al., 2016).

LPS robustly induces NET formation. NETs strongly promote thrombosis through multiple mechanisms. First, their net-like structure provides a physical scaffold for platelet adhesion, red blood cell aggregation, and fibrin deposition. Second, their core component, histones, possess direct cytotoxicity and procoagulant activity. NETs can also amplify the intrinsic and extrinsic coagulation pathways by activating platelets and enhancing thrombin generation. *In vitro* studies have confirmed that NETs can aggregate platelets and induce the formation of red blood clots (Zhang et al., 2023).

Metabolic products of the intestinal microbiota, such as TMAO, are independent risk factors for thrombosis. TMAO can enhance calcium ion signaling in platelets, increasing their responsiveness to various agonists and promoting platelet aggregation (Witkowski et al., 2022). Additionally, TMAO can promote vascular inflammation by activating the NF-κB and NLRP3 inflammasome signaling pathways in vascular endothelial cells, jointly creating a microenvironment conducive to thrombosis (Seldin et al., 2016).

### 3.6 Role of gut microbiota in the kidneys during sepsis

The translocation of intestinal microbiota contributes to the sepsis-associated acute kidney injury (AKI). This microbial translocation causes kidney damage by triggering a systemic inflammatory storm, releasing specific metabolic toxins, and interfering with the host’s immune metabolism.

#### 3.6.1 Inflammatory mediator damage and microcirculation disorders

After the disruption of the intestinal barrier, large amounts of LPS and other substances enter the circulation, activating signaling pathways such as TLR/NF-κB and triggering the release of cytokines like TNF-α, IL-1β, and IL-6, which leads to a cytokine storm. These mediators are carried by the blood flow to the kidneys, where they induce apoptosis of renal tubular epithelial and endothelial cells. Immune cells, including neutrophils, are then recruited to infiltrate the kidney tissue, amplifying local inflammation. This inflammation further causes vascular imbalance and endothelial damage, and abnormal renal hemodynamics, ultimately leading to renal ischemia and microthrombosis.

#### 3.6.2 Toxicity of metabolites

Disruption of the microbiota alters the metabolic product spectrum, exacerbating renal dysfunction. This disruption increases the production of toxins; specifically, intestinal bacteria convert choline and other substances into trimethylamine, which is oxidized by the liver to TMAO, a metabolite that promotes microthrombosis and generates uremic toxins such as indoxyl sulfate, thereby directly damaging the renal tubular interstitium (Bao et al., 2022). Concurrently, protective products decrease; for example, the production of beneficial SCFAs, such as butyric acid, is insufficient. It is important to note that these correlations were primarily observed in patients with chronic kidney disease (Corte-Iglesias et al., 2025). In sepsis-associated acute kidney injury, the timeframe and pathophysiology differ; thus, direct extrapolation should be made with caution. However, the potential for SCFA depletion to worsen renal injury in sepsis remains a plausible hypothesis that warrants dedicated clinical studies. Similarly, patients with immunoglobulin A nephropathy also exhibit decreased levels of SCFAs, especially butyric and propionic acid, and these metabolite levels are significantly negatively correlated with renal injury markers (Chai et al., 2021). Butyric acid has multiple protective effects, including anti-inflammatory actions, epigenetic regulation, and the maintenance of kidney protective factors, such as Klotho expression. Its deficiency not only compromises intestinal barrier repair but also disrupts direct kidney protection (Favero et al., 2024).

#### 3.6.3 Reprogramming of immune metabolism

Intestinal-derived signals can remotely affect renal immune cells. In the AKI environment, T cells infiltrating the kidneys undergo metabolic reprogramming, characterized by enhanced glutamine metabolism that amplifies inflammation within the kidney. Animal models have confirmed that inhibiting this metabolic pathway can alleviate renal injury (Lee et al., 2023).

## 4 Conclusions

This review provides a comprehensive synthesis of the “gut-organ axis” in sepsis-induced multi-organ dysfunction, a framework that has not been systematically applied in previous reviews. By integrating evidence from both experimental models and clinical observations, we highlight key gaps, particularly the need to distinguish between sepsis-specific mechanisms and those extrapolated from chronic diseases such as Parkinson’s disease or hypertension. Critically, we emphasize the bidirectional nature of the gut-sepsis cycle: microbial translocation amplifies systemic inflammation, which further disrupts the intestinal barrier, creating a self-perpetuating loop. Addressing this loop through microbiota-targeted therapies—such as SCFA supplementation, probiotic restoration, or fecal microbiota transplantation—represents a promising avenue for breaking the cycle of organ failure. Future studies should prioritize clinical trials that incorporate microbiome modulation as an adjunct to standard sepsis care.
